# Tetralogy of Fallot in Spain: a nationwide registry-based mortality study across 36 years

**DOI:** 10.1186/s13023-019-1056-y

**Published:** 2019-04-08

**Authors:** Laura Llamosas-Falcón, Eva Bermejo-Sánchez, Germán Sánchez-Díaz, Ana Villaverde-Hueso, Manuel Posada de la Paz, Verónica Alonso-Ferreira

**Affiliations:** 1 Preventive Medicine and Public Health, Hospital Universitario 12 de Octubre, 28041 Madrid, Spain; 2 Institute of Rare Diseases Research (IIER), Instituto de Salud Carlos III, 28029 Madrid, Spain; 3 Centre for Biomedical Network Research on Rare Diseases (CIBERER), 28029 Madrid, Spain; 4 Department of Geology, Geography and Environmental Sciences, University of Alcala, 28801 Alcalá de Henares, Spain

**Keywords:** Congenital heart defect, Tetralogy of Fallot, Mortality, Spain, Temporal-analysis, Spatial-analysis

## Abstract

**Background:**

Tetralogy of Fallot (TOF) is the most frequent cyanotic congenital heart defect. TOF mortality has fallen remarkably in recent years due to therapeutic advances. Accordingly, the aim of this study was to assess temporal and spatial variability in TOF-related mortality in Spain across the period 1981–2016, using data drawn from the nationwide population-based registry.

**Methods:**

Annual deaths due to TOF were sourced from the Spanish National Institute of Statistics database by reference to International Classification of Diseases (ICD), 9th and 10th Revision codes, namely, ICD-9 code 745.2 (period 1981–1998) and ICD-10 code Q21.3 (period 1999–2016). Age-specific and age-adjusted mortality rates were calculated, as were standardised mortality ratios (SMRs) by province, district and municipality for the period 1999–2016.

**Results:**

A total of 1035 deaths were attributed to TOF (57.78% of them were men and 42.22% were women). The age-adjusted mortality rate ranged from 0.75 per 1,000,000 inhabitants (95% confidence interval [CI]: 0–1.36) in 1981 to 0.03 per 1,000,000 (95% CI: 0.01–0.06) in 2016 for both sexes. In 2011, there was a change in the mortality trend, with a significant decrease of 49.22% per year (*p* < 0.001). In terms of geographical analysis, some areas with a significantly higher risk of TOF mortality were identified in the south of Spain, though no specific spatial pattern was in evidence.

**Conclusion:**

The decrease in TOF mortality may be related to improvements in diagnostic and treatment techniques. More studies are needed to analyse regions with a higher mortality risk, in order to improve medical planning and resource allocation, and identify risk factors and preventive measures.

## Background

Tetralogy of Fallot (TOF) is a structural congenital heart defect characterised by the presence of four cardiac defects: infundibular stenosis of the pulmonary artery, overriding aortic root, ventricular septal defect, and right ventricle hypertrophy [1, 2]. TOF has an estimated world-wide prevalence of 3 cases per 10,000 live births and can thus be considered a *rare disease* [3, 4]. Despite its low prevalence, it ranks among the most frequent congenital heart defects (CHD). The global birth prevalence of CHD in the general population is 0.8%, and TOF represents 5–10% of all CHD [5, 6]. A CHD study conducted at a regional level in Spain confirmed that TOF is the most frequent cyanotic CHD, with a birth prevalence of 0.41% [7]. At a European level, EUROCAT (the European population-based consortium registry on congenital anomalies) data show a significant increase in TOF prevalence from 2004 to 2012 [8]. In contrast, a recent nationwide registry-based study in Spain reported decreasing trends in mortality rates due to congenital anomalies from 1999 to 2013 [9].

In the absence of treatment, TOF’s natural disease course leads to high morbidity and mortality [10]. Although *mortality* from this cause has been reduced as a consequence of the impact of surgical interventions, patients still have a high risk of premature death, as compared to the general population [11]. This serves to highlight the interest that lies in analysing and monitoring mortality directly related to TOF. Mortality analyses provide information on demographic change, and allow for socio-economic strategies to be designed with the aim of improving the health and well-being of the population [9, 12].

While several studies have specifically analysed TOF patient mortality following surgical repair of the defect [11, 13–15], others have focused on overall CHD mortality [16–18]. In addition, some other TOF studies report on long-term outcome and survival analyses [19–23].

As regards *geographical variability*, Garcia et al. [24] reported significant differences in the distribution of CHD cases with postnatal diagnosis in Colombia but failed to find any specific pattern in this distribution. In China, a progressive increase in the prevalence of atrial septal defect and patent ductus arteriosus was observed in areas of higher altitude [25]. A cluster of births with TOF was found in a largely agricultural area of North Carolina (USA) but there was insufficient evidence to conclude that this was due to any given environmental factor [26]. In Spain, a temporal and spatial analysis of CHD prevalence was carried out in the Valencian Region (*Comunidad Valenciana*) in the east of Spain, where the authors found a heterogeneous geographic pattern [27]. In a recent study mentioned above [9], a high risk of premature death due to congenital anomalies was specifically identified in the south of Spain but, apart from this analysis, no population-based studies on TOF-attributed mortality in Spain have been reported to date, and there is no nationwide study on the subject, all of which make the present study unique.

Accordingly, the aim of this study was to analyse the mortality trends due to TOF in Spain over a lengthy period of time spanning 36 years (1981–2016), in order to assess whether there has been some kind of trend, and to try and discern possible geographical differences in the risk of death attributed to this disease.

## Results

A total of 1035 TOF-related deaths were identified in Spain during the period 1981–2016 (631 from 1981 to 1998, and 404 from 1999 to 2016), 57.78% of which were males (598 males vs. 437 females). The mean *age of death* was 16.28 ± 19.41 years and the median was 5 years, with no statistically significant differences between the sexes (*p* = 0.100). In both sexes combined, the age groups “*less than 1 year”* and “*1–4 years”* registered the highest percentage of deaths (19 and 29.5% respectively), meaning that 48.5% of the 1035 TOF deaths occurred before the age of 5 years.

**Fig. 1:**
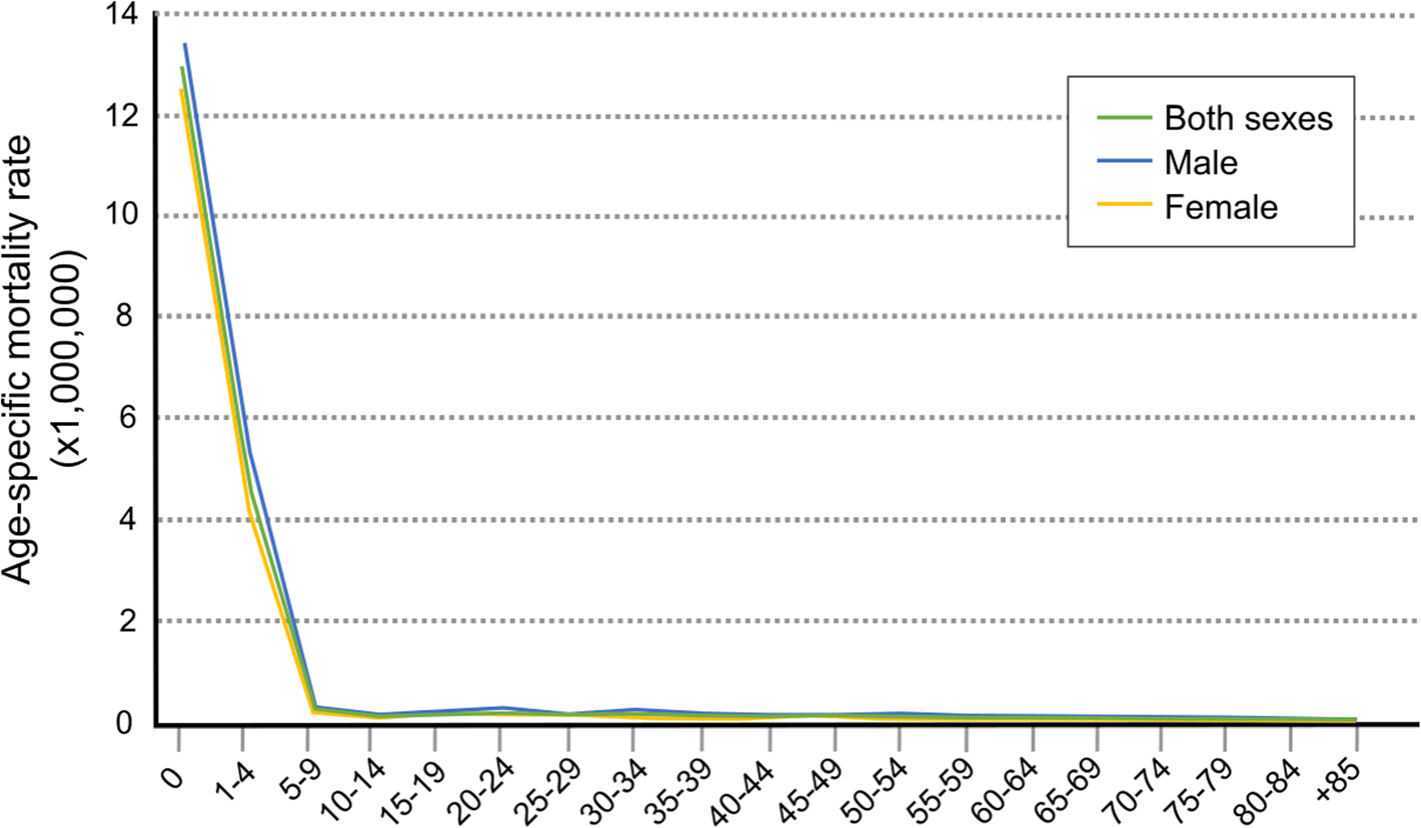
Annual age-specific mortality rates attributed to Tetralogy of Fallot by sex

Figure 1 depicts the *age-specific mortality rates*, with the mortality rate being highest among children under one year of age (12.98 per 1,000,000) and then dropping sharply after 5 years of age (0.26 per 1,000,000). The same pattern was observed for both males and females.

**Fig. 2:**
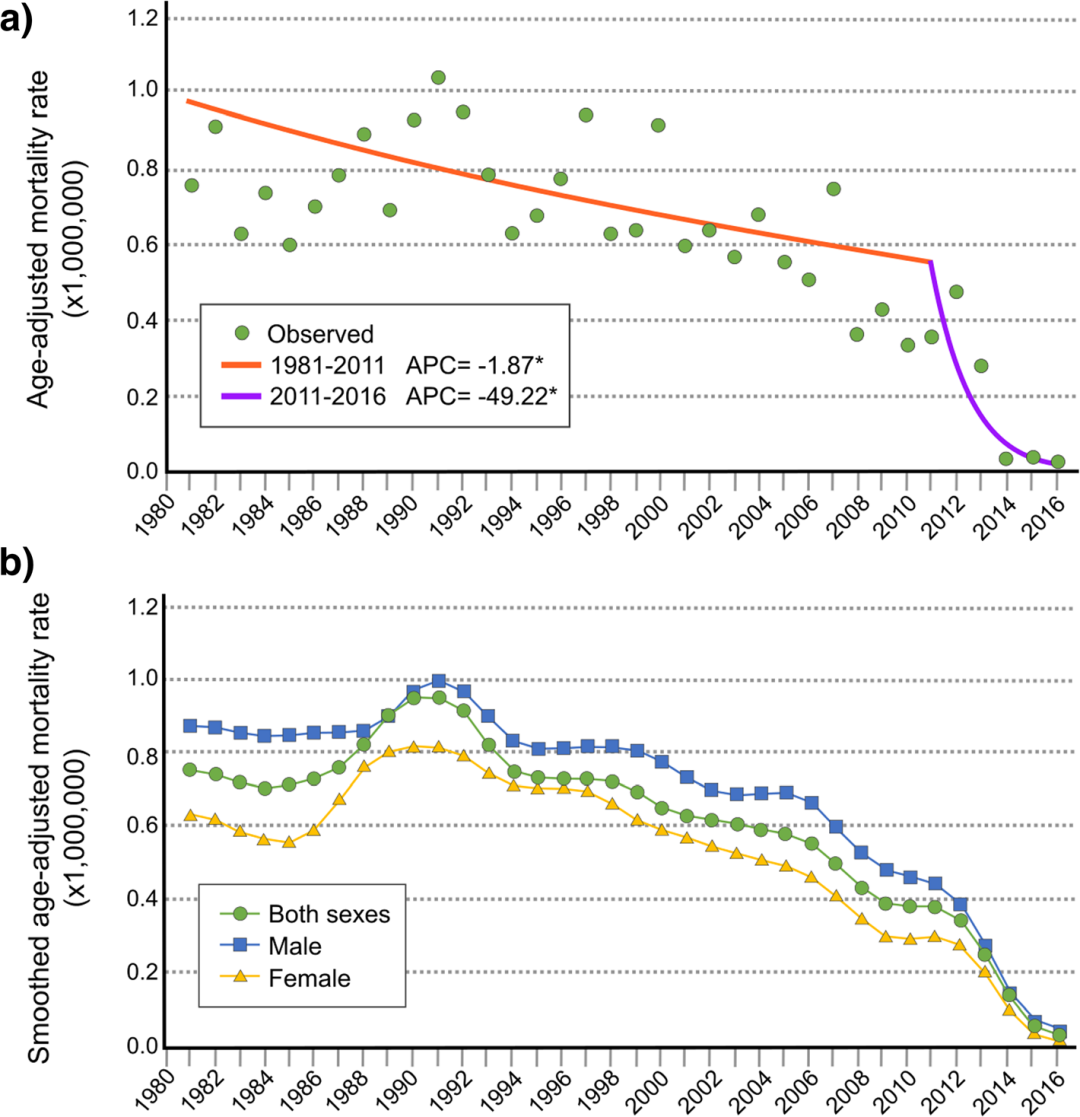
Age-adjusted mortality rates due to Tetralogy of Fallot in Spain, 1981–2016. **a** Mortality trend plotted using a Joinpoint regression model; **b** Smoothed annual rates by sex

In terms of the *trend over time*, the crude TOF mortality rate was seen to drop from 1.11 per 1,000,000 in 1981 to 0.03 in 2016. The *age-adjusted mortality rate*, likewise for both sexes, decreased from 0.75 per 1,000,000 inhabitants (95% confidence interval [CI]: 0–1.36) in 1981 to 0.03 per 1,000,000 (95% CI: 0.01–0.06) in 2016 (non-smoothed values). The Joinpoint model (Fig. 2a) showed that there was no significant trend in the adjusted rates from 1981 to 2010 but that from 2011 onwards there was a decrease of 49.22% per year (*p* < 0.001). The trend in age-adjusted rates is shown for each sex in Fig. 2b (smoothed values). In recent decades, a progressive decrease was in evidence, with the trend in smoothed rates being similar for both sexes, whether considered jointly or separately.

**Fig. 3:**
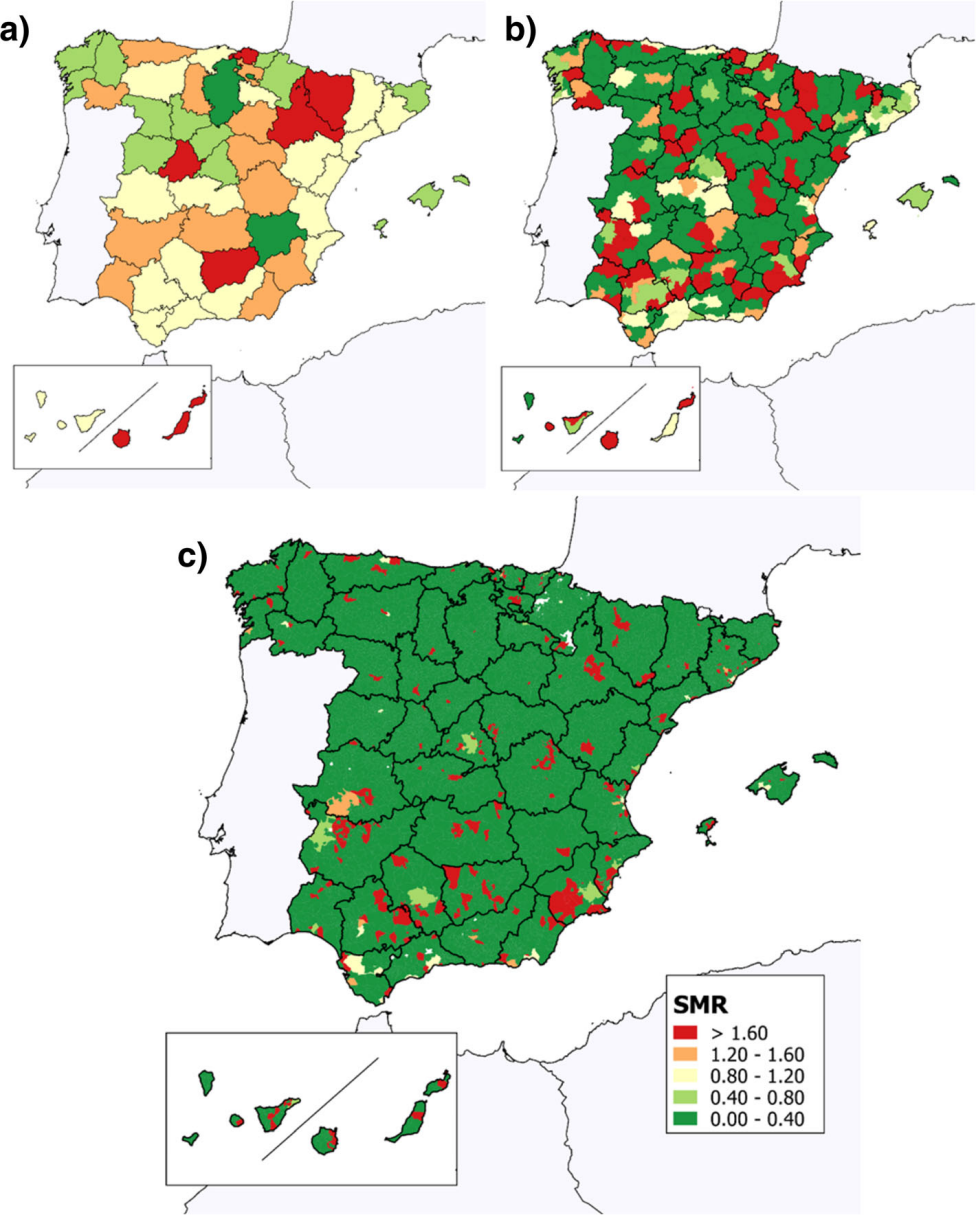
Geographical representation of standardised mortality ratios (SMRs) for tetralogy of Fallot in Spain, 1999–2016. **a** Provinces; **b** Districts; **c** Municipalities

Insofar as the geographical analysis is concerned, the variability in SMRs across the period 1999–2016 is depicted in Fig. 3 with a breakdown by province, district and municipality. A heterogeneous distribution is observed without any evident defined spatial pattern. It would, however, seem that regions with SMRs higher than 1 are located mainly, though not exclusively, in areas of southern Spain. When the TOF mortality in adjacent regions (smoothed data not shown) was taken into account, the variability detected in the smoothed model was completely blurred, with no differences among municipal results.

Table 1 shows the provinces, districts and municipalities with SMRs significantly above or below 1 for the period 1999–2016. At a provincial level, whereas mortality in Madrid was significantly lower than expected (for both sexes combined and for males alone), it was significantly higher than expected (for both sexes) in Las Palmas. At a district level, the risk of mortality for both sexes combined was lower than expected in different districts of the province of Madrid (“Sur Occidental” and “Área Metropolitana”), and higher than expected in Las Palmas (“Gran Canaria”), Seville (“La Sierra Sur”) and Murcia (“Suroeste”). Among females, the SMR was also higher than expected in one district of the province of Murcia (“Campo de Cartagena”), while among males the Madrid district of “Area Metropolitana” was identified as having a lower-than-expected SMR.

**Table 1:** Standardised mortality ratios (SMRs) by province, district and municipality in 1999–2016, and 95% confidence intervals, global and by sex. Only those with statistically significant lower of higher-than-expected SMRs after indirect standardisation are shown

Risk	Location	Province	District	Municipality^b^	Both sexes	Male	Female
Low risk	C	Madrid			0.63 (0.43–0.89)	0.49 (0.27–0.75)	
	C	Madrid	Sur Occidental		0 (0.00–0.59)	0 (0.00–0.99)	
	C	Madrid	Area Metropolitana			0.52 (0.26–0.93)	
	C	Madrid	Madrid	Horcajuelo de la Sierra		0.31 (0.10–0.74)	
High risk	SW^a^	Las Palmas			1.80 (1.05–2.89)		
	SW^a^	Las Palmas	Gran Canaria		1.92 (1.05–3.22)		
	SW	Seville	Sierra Sur		5.09 (1.02–14.86)		
	SE	Murcia	Suroeste		2.88 (1.05–6.27)		
	SE	Murcia	Campo de Cartagena				3.90 (1.26–9.11)
	NE	Barcelona	Osona	Gurb	2.79 (1.02–6.07)		4.65 (1.25–11.90)
	N	Vizcaya	Vizcaya	Arrankudiaga	2.84 (1.22–5.59)	3.61 (1.32–7.87)	
	SE	Murcia	Suroeste	Aledo	3.03 (1.01–6.59)		5.10 (1.37–13.05)
	SW	Cadiz	La Janda	Barbate	3.85 (1.04–9.87)		
	C	Madrid	Campiña	Loeches	9.44 (1.06–34.07)		23.71 (2.66–85.60)
	E	Castellon	Llanos Centrales	Benasal		10.23 (1.15–36.92)	
	N	Palencia	Campos	Villovieco		10.41 (1.17–37.58)	
	SW^a^	Las Palmas	Fuerteventura	Oliva (La)	11.06 (1.30–41.88)		
	E	Valencia	Sagunto	Segart		13.97 (1.57–50.44)	
	NE	Barcelona	Osona	Tona	14.98 (1.68–54.08)		

^a^Island territories (Canary and Balearic Islands)

C = Centre; E = east; N = north; NE = north-east; NW = north-west; S = south; SE = south-east; SW = south-west; W = west

^b^Small significant municipalities are not displayed because of the numerical instability (population < 5000 inhabitants and SMR > 20)

In the case of municipalities, as many as 27 had significantly higher risks of premature death, when both sexes were jointly analysed. Taking males and females separately, the number of municipalities with SMRs significantly higher than 1 was 22 and 13 respectively, and only one municipality had an SMR significantly lower than 1, in men. These municipalities were distributed throughout the territory, without displaying any specific spatial grouping.

## Discussion

This nationwide study, which is the first to analyse mortality directly related to TOF in Spain over a period of 36 years, detected a significant decrease in mortality rates since 2011 and a higher-than-expected risk of death in certain regions of the country.

This observed decline over time in mortality can be accounted for by several factors. Advances in prenatal diagnosis, that have enabled earlier detection of foetal anomalies [23, 28–31], specifically in Spain [32–34], plus advances in surgical intervention techniques [13, 14, 20, 35], also in Spain [36], are the main factors identified as being responsible for the decrease in mortality. In addition, this downturn has also been influenced by the implementation of and improvement in cardiac rehabilitation programmes [37–39] coupled with ambulatory management and follow-up [40].

An increase in the prevalence of CHD in the period 2004–2012 was detected in EUROCAT, the population-based registry of congenital anomalies, and attributed to the proliferation of the main risk factors associated with CHD [8]. Similarly, prevalence of CHD was observed to increase in the Valencian Region across the period 1999–2008, possibly due to improvements in diagnostic techniques [27]; and indeed, the spread in the use of such techniques might also account for part of this same rise. According to our data, the increase in prevalence observed by these studies has not triggered an increase in absolute TOF mortality figures in Spain, as might have been expected. While elective terminations of pregnancy due to foetal anomalies (ETOPFA) are registered by EUROCAT, they do not appear in our analysis because NSI mortality figures do not include ETOPFA or stillbirths. In all probability, ETOPFA have contributed to the birth of a higher proportion of less severely affected cases, with a consequently lower risk of associated mortality [41, 42].

With regard to early diagnosis, in some cases this congenital anomaly can only be diagnosed postpartum. All intrauterine imaging methods have their limitations, despite the fact that there have been significant advances in these techniques [30]. Moreover, the prognosis for TOF cases detected in the prenatal period is worse than for those identified in the postnatal period, probably because the former tend to be more severely affected and this leads to their early detection. In fact, a higher incidence of chromosomal abnormalities, extra cardiac malformations and complex forms of TOF are usually associated with cases detected in the prenatal stage [29, 31]. Despite this, early diagnosis provides benefits when it comes to maintaining a close follow-up of the pregnancy [43]. This is also true for postnatally detected cases. The recent use of pulse oximetry for the screening of critical CHD is capable of detecting around six infants having a critical CHD such as TOF, among 10,000 apparently healthy newborn infants screened [44], and this will surely have an impact on morbidity and mortality in future analyses.

Age-specific mortality rates are higher in the first year of life and up to 5 years of age, decreasing thereafter with increasing age. It is important to take this information into account because it allows for those in charge of medical care to focus a special follow-up on this specific age range and monitor the different determinants of early death [9].

The number of patients reaching adult ages is in large part determined by the mortality arising from the surgery required in some cases, and by the increase of post-surgery survival over the years [13, 20, 22, 45–48]. This has given rise to an increasing number of surviving adults with complex CHD. Nevertheless, these patients still face premature death when they are young adults, with the causes of death mainly being heart failure or sudden death [17, 49]. The excess mortality in young adults as compared to the general population renders close specialist life-long follow-up of this group of patients advisable [17, 18, 50, 51].

In connection with age of death, studies suggest that, in an elderly population, the medical conditions of comorbidity acquired throughout life, including heart failure, diabetes, other chronic diseases and cancer, as well as advanced age *per se*, have a greater impact on mortality than suffering from a CHD [16, 52]. This could also influence the lower risk observed by us of TOF-related death at older ages, for which there might well be a predominance of the above-mentioned causes.

In terms of geographical analysis, while some provinces show a lower-than-expected risk, a series of significantly higher-than-expected risks of TOF-related mortality were observed in some demarcations, mostly in southern Spain. Even so, the data analysed do not fit any definite spatial country-wide pattern. The study conducted by Nelson et al. in North Carolina (USA) showed a cluster of TOF cases in mainly agricultural areas but did not identify any specific environmental risk factors [26]. In their spatial analysis of CHD prevalence in the Valencian Region [27], the authors also found a heterogeneous geographical distribution which they felt might be due to differences in diagnostic accuracy and coding at hospitals. A recent nationwide registry-based study reported a higher risk of death attributed to congenital anomalies in the south of Spain [9]. Although the Spanish National Health System (SNHS) guarantees equal access to basic health services, variability between provinces might be related to different determinants, such as access to highly specialised facilities, longer or shorter follow-up of patients, or parents’ attitudes to interruption of pregnancies in the case of foetuses affected with severe congenital heart defects. The data presented here did not allow for inference of any common local characteristics shared by the affected areas, but they constitute a body of evidence which should prompt new analytic studies and thereby allow possible links to be established between the disease’s distribution patterns and possible underlying environmental risk factors. This would generate new evidence and could lead to prevention at different levels.

As for measures that could reduce CHD mortality, one would be the designation of referral centres specialised in the follow-up, care and treatment of these anomalies. Other authors have observed that neonatal mortality increases when fewer patients are treated at specialised centres [53]. In Sweden, centralisation of cases in institutions specialised in paediatric cardiac surgery reduced mortality in patients who underwent surgical interventions [54]. These specialised hospitals helped to adopt new treatment strategies in more complex cases and channel information about such strategies to local hospitals more easily. In addition, the policy served to stimulate interest in the field and direct research activities on paediatric diseases with surgical treatment. In Spain, a similar designation exists in the shape of SNHS Referral Centres, Services and Units (*Centros, Servicios y Unidades de Referencia/CSUR*). Our data might make it advisable to review patients’ access to specific CSUR and their distribution across the country.

The limitations of this study include those usually attributed to death statistics, in which the underlying cause of death may be underestimated due to errors of diagnosis or coding, or to deaths being assigned to secondary complications instead of the disease itself, i.e., TOF. In addition, ETOPFA and stillborn cases were not included in our data (as vital statistics in Spain do not include these). Furthermore, the milder forms of TOF (with a lower mortality risk) can escape detection until older ages, potentially biasing the results towards more severe subtypes and higher overall mortality figures at younger ages.

Despite these limitations, the strength of this study lies in providing population-based information about officially reported deaths associated with this rare disease in Spain, over the span of 36 years. Its homogeneous, standardised and continuous methodology over time, allows this study contributing to monitoring mortality directly associated with TOF.

## Conclusions

In conclusion, this is the first nationwide population-based study to analyse the time trend and spatial variability of TOF-related mortality in Spain. A decrease in mortality since 2011 was detected, as well as a heterogeneous distribution throughout the country. This study not only shows the utility of spatial modelling using population mortality data, but can also contribute to future studies focusing on environmental and even epigenetic risk factors. Lastly, it gives a global view of the country, which could be used for further analyses on access to specialised care and other health services, ranging from pre- and neonatal screening and surgery at early ages, to long-term care for adults or elderly living with CHD such as TOF. Our finding of a decrease in TOF-related mortality -and thus more persons surviving into adulthood- means that those reaching reproductive ages will need to be adequately informed about their risk of having affected offspring, information that can be provided by specialists in Genetics. Higher survival at adult ages also implies the need to understand the clinical course of this disorder, in order to provide optimal medical care and anticipate possible complications, with the aim of equalising life expectancy vis-à-vis that of the general population.

## Methods

We designed and conducted an observational, retrospective, descriptive study. Annual deaths registered as due to TOF were sourced from the National Statistics Institute (NSI) of Spain. Deaths attributed to TOF as primary cause of death were identified by reference to International Classification of Diseases (ICD), 9th and 10th Revision codes for the underlying cause of death, namely, ICD-9 code 745.2 for the period 1981–1998 and ICD-10 code Q21.3 for the period from 1999 onwards.

In addition to collecting data on age, sex, year of birth, place of residence, registered year and place of death, we also obtained annual Spanish population data, broken down by sex, age and place of residence from the NSI, which we used to calculate annual sex- and age-group specific rates for each of the years studied.

We calculated the mean age at death, and compared the median age of death of men and women using the Mann-Whitney U test. Age-specific mortality rates and annual age-adjusted mortality rates were calculated by sex (direct standardisation using the standard European population as reference). The age-adjusted mortality rates were smoothed according to the non-parametric method T4253H [55] and the time trends were assessed using the Joinpoint regression model. All rates were expressed per 1,000,000 inhabitants.

The study area encompassed all of Spanish territory, made up of a total of 52 provinces, 326 districts and 8112 municipalities. For comparability reasons, geographical analysis considered the period 1999–2016 in which municipality identification was available in all TOF deaths. Standardised mortality ratios (SMRs) were calculated by province, district and municipality using the Spanish population as a reference (indirect method), with these data also being furnished by the NSI. The SMRs were subsequently smoothed taking into account information pertaining to adjacent geographical units, in accordance with the model proposed by Besag, York and Molliè [56]. No additional data transformation was made.

All statistical analyses were performed using the SPSS v15 (IBM Corporation, Chicago, IL, USA), EPIDAT v4.2 (General Directorate of Public Health, Galicia, Spain), Joinpoint v4.5.0.1 (National Cancer Institute, Bethesda, MD, USA) and R-INLA (Norwegian University of Science and Technology, Trondheim, Norway) computer software programmes, while QGIS v2.18.17 was used for cartographic representation purposes.

## Abbreviations

CHD: Congenital heart defects
CI: Confidence interval
ETOPFA: Elective terminations of pregnancy due to foetal anomalies
ICD: International Classification of Diseases
NSI: National Statistics Institute
SMR: Standardised mortality ratio
SNHS: Spanish National Health System
TOF: Tetralogy of Fallot
